# Supplementary material for: A dual endosymbiosis supports nutritional adaptation to hematophagy in the invasive tick Hyalomma marginatum
Source: eLife. 2021 Dec 24;10:e72747. doi: 10.7554/eLife.72747 (PMC8709577; doi:10.7554/eLife.72747)
Supplement: Supplementary file 1. — Supplementary file 1 presents as well the genes and the primers used for qPCR assays conducted for relative quantification of FLE and Midichloria. [file elife-72747-supp1.docx]

**SUPPLEMENTARY FILE 1**

**A dual endosymbiosis supports nutritional adaptation to hematophagy in the invasive tick *Hyalomma marginatum***

Buysse, Floriano *et al.*

**Supplementary files**

**Supplementary File 1a. Summary of the assembly information and quality analyses of the newly sequenced genomes of FLE, *Midichloria* and *Rickettsia* bacteria.** Only genomes with high completeness are shown and the partial genome of *R. aeaschlimannii* (RAES-Hmar-IS) was not presented.

| **Specimen details** | | | **Assembling information** | | | | | | | | |  |
| --- | --- | --- | --- | --- | --- | --- | --- | --- | --- | --- | --- | --- |
| Genome | Isolate | Origin | Genome size (bp) | Contigs number | N50 | L50 | Completeness (%) | GC rate (%) | Genes | Pseudogenes | Coding density (%) | Coverage values |
| *Francisella-*LE of *Hyalomma marginatum* | FLE-Hmar-ES | Spain | 1510123 | 17 | 254696 | 3 | 0,9905 | 31,11 | 937 | 795 | 54 | 733 |
| *Francisella-*LE of *Hyalomma marginatum* | FLE-Hmar-IT | Italy | 1510130 | 13 | 382079 | 2 | 0,9905 | 31,11 | 934 | 789 | 54 | 950 |
| *Francisella-*LE of *Hyalomma marginatum* | FLE-Hmar-IL | Israel | 1536710 | 25 | 106485 | 5 | 0,9905 | 31,23 | 961 | 798 | 54 | 92 |
| *Midichloria* of *Hyalomma marginatum* | Midi-Hmar-ES | Spain | 1168604 | 179 | 19210 | 20 | 0,981 | 35,03 | 1071 | 192 | 78 | 648 |
| *Midichloria* of *Hyalomma marginatum* | Midi-Hmar-IT | Italy | 1130547 | 359 | 10359 | 30 | 0,9524 | 35,23 | 1032 | 192 | 76 | 80 |
| *Midichloria* of *Hyalomma marginatum* | Midi-Hmar-IL | Israel | 1144281 | 123 | 12627 | 30 | 0,981 | 35,03 | 1055 | 190 | 79 | 63 |
| *Rickettsia aeschlimannii* (of *H. marginatum*) | RAES-Hmar-IT | Italy | 1356794 | 165 | 67640 | 4 | 0,9429 | 32,32 | 1122 | 475 | 68 | 563 |

**Supplementary File 1b. Summary of the information on the NCBI reference genomes employed for the characterization of the FLE and *Midichloria* genomes.**

| **Specimen details** |  |  |  | |  | **Assembling information** | | |  |
| --- | --- | --- | --- | --- | --- | --- | --- | --- | --- |
| Genome | Isolate | Origin | Accession number (NCBI/dryad) | Reference | | Genome size (bp) | Contigs number | GC rate (%) | Coding density (%) |
| *Francisella-*LE of *Ornithodoros moubata* | FLEOm | Lab colony (Czech Republic) | LVCE00000000 | Gerhart et al., 2018 | | 1564190 | 8 | 31,7 | 58 |
| *Francisella-*LE of *Ornithodoros moubata* | FOm | Lab colony (France) | QAPC00000000 | Duron et al., 2018 | | 1566493 | 11 | 31,7 | 57 |
| *Francisella-*LE of *Amblyomma maculatum* | FLEAm | United States of America | LNCT00000000 | Gerhart et al., 2018 | | 1556261 | 7 | 31,67 | 57 |
| *Francisella-*LE of *Argas arboreus* | *F. persica* (ATCC VR-331) | Egypt | CP013022 | Larson et al., 2016 | | 1540768 | 1 | 31,43 | 69 |
| *Francisella-*LE of *Hyalomma asiaticum* | NMGha432 | China | doi.org/10.5061/dryad.t76hdr80p | Buysse and Duron, 2021 | | 1550715 | 38 | 31,62 | 58 |
| *Francisella-*LE of *Hyalomma asiaticum* | XJHA498 | China | doi.org/10.5061/dryad.t76hdr80p | Buysse and Duron, 2021 | | 1549389 | 39 | 31,6 | 58 |
| *Francisella tularensis* (tularemia agent) | SCHU-S4 | United States of America | AJ749949 | Larsson et al., 2005 | | 1892775 | 1 | 32,26 | 83 |
| *Midichloria* of *Ixodes ricinus* | IricVA | Italy | CP002130 | Sassera et al., 2011 | | 1183732 | 1 | 36,55 | 71 |

**Supplementary File 1c. Details regarding symbionts used in phylogenetic analyses of B vitamin biosynthetic pathways and streamlined biotin operon of FLE and *Midichloria*.**

| **Specimen details** |  |  |  |  |  |  |  | **Biotin gene origin** | |
| --- | --- | --- | --- | --- | --- | --- | --- | --- | --- |
| Class | Order | Family | Genus/Species | Strain | Host species | Accession number | Reference | Horizontal transfer | Ancestral |
| Gammaproteobacteria | Thiotrichales | Francisellaceae | *Francisella*-like | FLE-Hmar-IL | Hard tick, *Hyalomma marginatum* (Israel) | - | This study |  | Lost |
|  |  |  | *Francisella*-like | FLE-Hmar-ES | Hard tick, *Hyalomma marginatum* (Spain) | - | This study |  | Lost |
|  |  |  | *Francisella*-like | FLE-Hmar-IT | Hard tick, *Hyalomma marginatum* (Italy) | - | This study |  | Lost |
|  |  |  | *Francisella*-like | NMGha432 | Hard tick, *Hyalomma asiaticum* (China) | - | Buysse and Duron, 2021 |  | + |
|  |  |  | *Francisella*-like | XJHA498 | Hard tick, *Hyalomma asiaticum* (China) | - | Buysse and Duron, 2021 |  | + |
|  |  |  | *Francisella*-like | FLEAm | Hard tick, *Amblyomma maculatum* | LNCT00000000 | Gerhart et al., 2018 |  | + |
|  |  |  | *Francisella*-like | FLEOm | Soft tick, *Ornithodoros moubata* | LVCE00000000 | Gerhart et al., 2018 |  | + |
|  |  |  | *Francisella*-like | FOm | Soft tick, *Ornithodoros moubata* | QAPC00000000 | Duron et al., 2018 |  | + |
|  |  |  | *Francisella*-like | *F. persica* | Soft tick, *Argas arboreus* | CP013022 | Suitor and Weiss, 1961 |  | + |
|  | Legionellales | Coxiellaceae | *Coxiella*-like | CRt | Hard tick, *Rhipicephalus turanicus* | CP011126 | Gottlieb et al., 2015 |  | + |
|  |  |  | *Coxiella*-like | CRm | Hard tick, *Rhipicephalus microplus* | NSHJ01000000 | Guizzo et al., 2017 |  | + |
|  |  |  | *Coxiella*-like | CLEAA | Hard tick, *Amblyomma americanum* | CP007541 | Smith et al., 2015 |  | + |
|  |  |  | *Coxiella*-like | CeAS-UFV | Hard tick, *Amblyomma sculptum* | CP033868 | Direct submission on GenBank (Vidigal et al.) |  | + |
|  |  |  | *Coxiella burnetii* | RSA493 | - | AE016828 | Seshadri et al., 2003 |  | + |
|  |  | Legionellaceae | *Legionella polyplacis* | PsAG | Louse, *Polyplax serrata* | CP021497 | Říhová et al., 2017 | + |  |
|  |  |  | *Legionella pneumophila* | Lens | - | CR628337 | Cazalet et al., 2004 |  | + |
|  | Enterobacterales | Morganellaceae | *Arsenophonus* endosymbiont | CB | Deer ked, *Lipoptena fortisetosa* | CP013920 | Direct submission on GenBank (Novakova et al.) |  | + |
|  |  |  | *Arsenophonus triatominarum* | ATi | Kissing bug, *Triatoma infestans* | LWMI00000000 | Direct submission on GenBank (Darby) |  | + |
|  |  |  | *Providencia siddallii* | officinalis | Leech, *Haementeria officinalis* | CVRF00000000 | Direct submission on GenBank (Manzano-Marin) |  | + |
|  |  | Erwiniaceae | *Buchnera aphidicola* | LSR1 | Aphid, *Acyrthosiphon pisum* | ACFK00000000 | Direct submission on GenBank (Shigenobu et al.) |  | + |
|  |  |  | *Buchnera aphidicola* | - | Aphid, *Myzus persicae* | MJNC00000000 | Direct submission on GenBank (Mathers et al.) |  | + |
|  |  |  | *Wigglesworthia glossinidia* | Yale colony | Tsetse fly, *Glossina morsitans* | CP003315 | Rio et al., 2012 |  | + |
|  |  |  | *Riesia pediculischaeffi* | PTSK | Louse, *Pediculus schaeffi* | CP012839 | Direct submission on GenBank (Boyd) |  | + |
|  |  | Pectobacteriaceae | *Sodalis*-like endosymbiont | IL | Psyllid, *Bactericera trigonica* | RBZS00000000 | Ghosh et al., 2020 |  | + |
|  |  |  | *Sodalis*-like endosymbiont | Spu | Cicada, *Philaenus spumarius* | NKXM00000000 | Ankrah et al., 2018 |  | + |
|  |  |  |  |  |  |  |  |  |  |
| Alphaproteobacteria | Rickettsiales | Midichloriaceae | *Midichloria* | MID-Hmar-IL | Hard tick, *Hyalomma marginatum* (Israel) | - | This study | + |  |
|  |  |  | *Midichloria* | MID-Hmar-ES | Hard tick, *Hyalomma marginatum* (Spain) | - | This study | + |  |
|  |  |  | *Midichloria* | MID-Hmar-IT | Hard tick, *Hyalomma marginatum* (Italy) | - | This study | + |  |
|  |  |  | *Cand.* Midichloria mitochondrii | IricVA | Hard tick, *Ixodes ricinus* | CP002130 | Sassera et al., 2011 | + |  |
|  |  | Rickettsiaceae | *Rickettsia buchneri* | ISO7 | Hard tick, *Ixodes scapularis* | JFKF00000000 | Kurtti et al., 2015 | + |  |
|  |  |  | *Rickettsia* endosymbiont | REIS | Hard tick, *Ixodes scapularis* | ACLC00000000 | Gillespie et al., 2012 | + |  |
|  |  |  | *Rickettsia felis* | LSU-Lb | Booklouse, *Liposcelis bostrychophila* | JSEL00000000 | Gillespie et al., 2014 |  | + |
|  |  |  | *Rickettsia aeschlimannii* | - | - | CCER00000000 | Direct submission on GenBank (Urnite platform) |  | + |
|  |  | Anaplasmataceae | *Wolbachia* | wNleu | Bee, *Nomada leucophthalma* | LYUV00000000 | Direct submission on GenBank (Gerth and Bleidorn) | + |  |
|  |  |  | *Wolbachia* | wNfla | Bee, *Nomada flava* | LYUW00000000 | Direct submission on GenBank (Gerth and Bleidorn) | + |  |
|  |  |  | *Wolbachia* | wCle | Bed bug, *Cimex lectularius* | AP013028 | Nikoh et al., 2014 | + |  |
|  |  |  | *Wolbachia* | wStri | Planthopper, *Laodelphax striatellus* | MUIX00000000 | Ju et al., 2020 | + |  |
|  |  |  | *Wolbachia* | wLug | Planthopper, *Nilaparvata lugens* | MUIY00000000 | Ju et al., 2020 | + |  |
|  |  |  | *Wolbachia* | wCfeT | Flea, *Ctenocephalides felis* | CP051156 | Direct submission on GenBank (Driscoll et al.) | + |  |
|  |  |  | *Neorickettsia sennetsu* | Miyayama | - | CP000237 | Dunning Hotopp et al., 2006 | + |  |
|  |  |  | *Neorickettsia risticii* | Illinois | - | CP001431 | Lin et al., 2009 | + |  |
|  |  |  | *Neorickettsia helminthoeca* | Oregon | Salmonid fish | CP007481 | Direct submission on GenBank (Lin et al.) | + |  |
|  |  |  | *Neorickettsia sp.* | 179522 | - | LNGI00000000 | Direct submission on GenBank (Mitreva et al.) | + |  |
|  |  |  | *Ehrlichia ruminantium* | Welgevonden | - | CR767821 | Collins et al., 2005 |  | + |
|  |  |  | *Anaplasma marginale* | Florida | - | CP001079 | Visser et al., 1992 |  | + |
|  |  |  |  |  |  |  |  |  |  |
| Deltaproteobacteria | Desulfovibrionales | Desulfovibrionaceae | *Lawsonia intracellularis* | N343 | - | CP004029 | Sait et al., 2013 | + |  |
|  |  |  | *Lawsonia intracellularis* | PHE/MN1-00 | - | AM180252 | Direct submission on GenBank (Kaur et al.) | + |  |
|  |  |  | *Lawsonia intracellularis* | Fu/JPN | - | QNHO00000000 | Direct submission on GenBank (Nishikawa et al.) | + |  |
|  |  |  |  |  |  |  |  |  |  |
|  | Cytophagales | Amoebophilaceae | *Cardinium* | cSfur | Planthopper, *Sogatella furcifera* | CP022339 | Direct submission on GenBank (Zeng) | + |  |
|  |  |  | *Cardinium* | cEper1 | Wasp, *Encarsia pergandiella* | HE983995 | Penz et al., 2012 | + |  |
|  |  |  | *Cardinium* | cBtQ1 | Whitefly, *Bemisia tabaci* | CBQZ00000000 | Santos-Garcia et al., 2014 | + |  |

**Supplementary File 1d. Illustrative (non-exhaustive) studies investigating the presence of FLE and *Midichloria* symbionts across *Hyalomma* tick species.** +: presence; –: absence; ND: not determined.

| **Specimen details** | | **Detection details** | | | |
| --- | --- | --- | --- | --- | --- |
| Tick species | Geographic origin | Symbiont presence | | Detection method | Reference |
|  |  | FLE | *Midichloria* |  |  |
| *Hyalomma* | Israel | + | - | PCR | Azagi et al., 2017 |
| *aegyptium* | Anatolia, Mugla Province | + | ND | Metagenomic analysis | Brinkmann et al., 2019 |
|  | Qatar, Doha | ND | + | PCR | Barradas et al., 2020 |
|  | Bulgaria | + | ND | PCR | Ivanov et al., 2011 |
|  |  |  |  |  |  |
| *H. anatolicum* | Turkey | - | - | PCR | Duron et al., 2017 |
|  | Turkey | ND | + | PCR & qPCR | Cafiso et al., 2016 |
|  | Pakistan, Punjab Province | + | + | Bacterial barcoding | Adegoke et al., 2020 |
|  |  |  |  |  |  |
| *H. asiaticum* | China, Xinjiang Province | + | ND | PCR | Wang et al., 2018 |
|  | China | + | - | Metagenomic analysis | Buysse and Duron, 2021 |
|  |  |  |  |  |  |
| *H. dromedarii* | Israel | + | ND | PCR | Azagi et al., 2017 |
|  | Saudi Arabia | + | ND | PCR | Elbir et al., 2020 |
|  | Tunisia, Sousse | ND | + | PCR | Selmi et al., 2019 |
|  | Tunisia, Kebili | ND | - | PCR | Selmi et al., 2019 |
|  | Egypt | + | ND |  | Ghoneim et al., 2017 |
|  | United Arab Emirates, Al-Ain | + | ND | Bacterial barcoding | Perveen et al., 2020 |
|  | Palestine, Jericho | + | - | Metagenomic analysis | Ravi et al., 2018 |
|  | Saudi Arabia, Al Hofuf | + | - | PCR & bacterial barcoding | Elbir et al., 2019 |
|  | Saudi Arabia, Hail Province | + | - | Bacterial barcoding | Alreshidi et al., 2020 |
|  |  |  |  |  |  |
| *H. excavatum* | Israel | + | ND | PCR | Azagi et al., 2017 |
|  | Tunisia, Kebili | ND | - | PCR | Selmi et al., 2019 |
|  | Turkey, Mecitözu | + | + | PCR | Duron et al., 2017 |
|  | Turkey | ND | + | PCR & qPCR | Cafiso et al., 2016 |
|  | Egypt | ND | + | Reverse line blotting (RLB) | AL-Hosary et al., 2021 |
|  |  |  |  |  |  |
| *H. impeltatum* | Tunisia, Gabes | ND | + | PCR | Selmi et al., 2019 |
|  | Tunisia, Sousse | ND | + | PCR | Selmi et al., 2019 |
|  | Tunisia, Kebili | ND | - | PCR | Selmi et al., 2019 |
|  | Tunisia, Kairouan | ND | - | PCR | Selmi et al., 2019 |
|  | Tunisia, Tataouine | ND | - | PCR | Selmi et al., 2019 |
|  | Zimbabwe | + | - | PCR | Duron et al., 2017 |
|  |  |  |  |  |  |
| *H. lusitanicum* | Spain | + | + | Bacterial barcoding | Díaz-Sánchez et al., 2021 |
|  | Canaries, Veneguera | + | - | PCR | Duron et al., 2017 |
|  | Spain, Madrid | + | ND | PCR | Lopes de Carvalho et al., 2016 |
|  | Portugal | - | ND | PCR | Lopes de Carvalho et al., 2016 |
|  |  |  |  |  |  |
| *H. marginatum* | Israel | + | ND | PCR & FISH | Azagi et al., 2017 |
|  | Anatolia, Mugla Province | + | ND | Illumina HiSeq sequencing | Brinkmann et al., 2019 |
|  | France | + | + | PCR | Duron et al., 2017 |
|  | Italy, Sicilia | ND | + | PCR | Epis et al., 2008 |
|  | Ventotene Island (Mediterranean Sea) | ND | + | PCR | Di Lecce et al., 2018 |
|  | Bulgaria | + | ND | PCR | Ivanov et al., 2011 |
|  | Spain, Madrid | - | ND | PCR | Lopes de Carvalho et al., 2016 |
|  | Portugal | - | ND | PCR | Lopes de Carvalho et al., 2016 |
|  |  |  |  |  |  |
| *H. rufipes* | Israel | + | ND | PCR | Azagi et al., 2017 |
|  | China, Gansu Province | - | + | High-throughput sequencing of small RNAs | Luo et al., 2017 |
|  | Ethiopia, Didessa Valley | + | ND | PCR | Szigeti et al., 2014 |
|  |  |  |  |  |  |
| *H. truncatum* | Namibia | + | ND | PCR | Direct submission on GenBank (JF290387) |
|  | Italy, Sicilia | ND | + | PCR | Epis et al., 2008 |

**Supplementary File 1e. Genes and primers used in this study.** Primers were used for qPCR assays conducted for relative quantification of FLE and *Midichloria*.

^1^ PCR conditions: primers concentration 150 nM; thermal profile: 95 °C for 3 min, and 40 cycles at 95 °C for 15 sec, 55 °C for 20 sec, 72 °C for 10 sec; ^2^ PCR conditions: primers concentration 250 nM; thermal profile: 95 °C for 3 min, and 40 cycles at 95 °C for 15 sec, 58 °C for 30 sec; ^3^ PCR conditions: primers concentration 250 nM; thermal profile: 95 °C for 3 min, and 40 cycles at 95 °C for 15 sec, 58°C for 30 sec.

| **Gene** | **Hypothetical product** | **Primers (5'-3')** | **Tm (°C)** | **Fragment size** | **Reference** |
| --- | --- | --- | --- | --- | --- |
| *Hyalomma marginatum* | |  |  |  |  |
| *cal* | Calreticulin | F: TACCTCAAGCTGTTCGACTG | 53 | F/R: 132 bp^1^ | This study |
|  |  | R: GCCCTTGTAGTTGAAGATG |  |  |  |
|  |  |  |  |  |  |
| FLE |  |  |  |  |  |
| *rpoB* | DNA-directed RNA | F: TGTACCTATTGCTACGCCAG | 58 | F/R: 122 bp^2^ | This study |
|  | polymerase beta chain | R: GCTTACCAGTACGACCATC |  |  |  |
|  |  |  |  |  |  |
| *Midichloria* |  |  |  |  |  |
| *gyrB* | DNA gyrase B subunit | F: ATTTACATGTCAGGGAGTG | 58 | F/R: 155 bp^3^ | This study |
|  |  | R: CTACGAGTTATTGCTGCTC |  |  |  |

**Supplementary references**

Adegoke A, Kumar D, Bobo C, Rashid MI, Durrani AZ, Sajid MS, Karim S. 2020. Tick-borne pathogens shape the native microbiome within tick vectors. *Microorganisms* **8**:1–16. doi:10.3390/microorganisms8091299

AL-Hosary A, Răileanu C, Tauchmann O, Fischer S, Nijhof AM, Silaghi C. 2021. Tick species identification and molecular detection of tick-borne pathogens in blood and ticks collected from cattle in Egypt. *Ticks Tick Borne Dis* **12**. doi:10.1016/j.ttbdis.2021.101676

Alreshidi MM, Veettil VN, Noumi E, Del Campo R, Snoussi M. 2020. Description of microbial diversity associated with ticks *Hyalomma dromedarii* (Acari: Ixodidae) isolated from camels in Hail region (Saudi Arabia) using massive sequencing of 16S rDNA. *Bioinformation* **16**:602–610. doi:10.6026/97320630016602

Ankrah NYD, Chouaia B, Douglas E. 2018. The cost of metabolic interactions in symbioses between insects and bacteria with reduced genomes. *MBio* **9**:1–15. doi: 10.1128/mBio.01433-18

Azagi T, Klement E, Perlman G, Lustig Y, Mumcuoglu KY, Apanaskevich DA, Gottlieb Y. 2017. *Francisella*-like endosymbionts and *Rickettsia* species in local and imported *Hyalomma* ticks. *Appl Environ Microbiol* **83**:1–14. doi:10.1128/AEM.01302-17

Barradas PF, Lima C, Cardoso L, Amorim I, Gärtner F, Mesquita JR. 2020. Molecular evidence of *Hemolivia mauritanica*, *Ehrlichia spp.* and the endosymbiont *Candidatus* Midichloria mitochondrii in *Hyalomma aegyptium* infesting *Testudo graeca* tortoises from Doha, Qatar. *animals*. doi:10.3390/ani11010030

Brinkmann A, Hekimoǧlu O, Dinçer E, Hagedorn P, Nitsche A, Ergünay K. 2019. A cross-sectional screening by next-generation sequencing reveals *Rickettsia*, *Coxiella*, *Francisella*, *Borrelia*, *Babesia*, *Theileria* and *Hemolivia* species in ticks from Anatolia. *Parasites and Vectors* **12**:1–13. doi:10.1186/s13071-018-3277-7

Buysse M, Duron O. 2021. Evidence that microbes identified as tick-borne pathogens are nutritional endosymbionts. *Cell*. doi: 10.1016/j.cell.2021.03.053

Cafiso A, Bazzocchi C, De Marco L, Opara MN, Sassera D, Plantard O. 2016. Molecular screening for *Midichloria* in hard and soft ticks reveals variable prevalence levels and bacterial loads in different tick species. *Ticks Tick Borne Dis* **7**:1186–1192. doi:10.1016/j.ttbdis.2016.07.017

Cazalet C, Rusniok C, Brüggemann H, Zidane N, Magnier A, Ma L, Tichit M, Jarraud S, Bouchier C, Vandenesch F, Kunst F, Etienne J, Glaser P, Buchrieser C. 2004. Evidence in the *Legionella pneumophila* genome for exploitation of host cell functions and high genome plasticity. *Nat Genet* **36**:1165–1173. doi:10.1038/ng1447

Collins NE, Liebenberg J, De Villiers EP, Brayton KA, Louw E, Pretorius A, Faber FE, Van Heerden H, Josemans A, Van Kleef M, Steyn HC, Van Strijp MF, Zweygarth E, Jongejan F, Maillard JC, Berthier D, Botha M, Joubert F, Corton CH, Thomson NR, Allsopp MT, Allsopp BA. 2005. The genome of the heartwater agent *Ehrlichia ruminantium* contains multiple tandem repeats of actively variable copy number. *Proc Natl Acad Sci U S A* **102**:838–843. doi:10.1073/pnas.0406633102

Di Lecce I, Bazzocchi C, Cecere JG, Epis S, Sassera D, Villani BM, Bazzi G, Negri A, Saino N, Spina F, Bandi C, Rubolini D. 2018. Patterns of *Midichloria* infection in avian-borne African ticks and their trans-Saharan migratory hosts. *Parasites and Vectors* **11**:1–11. doi:10.1186/s13071-018-2669-z

Díaz-Sánchez S, Fernández AM, Habela MA, Calero-Bernal R, de Mera IGF, de la Fuente J. 2021. Microbial community of *Hyalomma lusitanicum* is dominated by *Francisella*-like endosymbiont. *Ticks Tick Borne Dis* **12**. doi:10.1016/j.ttbdis.2020.101624

Dunning Hotopp JC, Lin M, Madupu R, Crabtree J, Angiuoli S V., Eisen J, Seshadri R, Ren Q, Wu M, Utterback TR, Smith S, Lewis M, Khouri H, Zhang C, Niu H, Lin Q, Ohashi N, Zhi N, Nelson W, Brinkac LM, Dodson RJ, Rosovitz MJ, Sundaram J, Daugherty SC, Davidsen T, Durkin AS, Gwinn M, Haft DH, Selengut JD, Sullivan SA, Zafar N, Zhou L, Benahmed F, Forberger H, Halpin R, Mulligan S, Robinson J, White O, Rikihisa Y, Tettelin H. 2006. Comparative genomics of emerging human ehrlichiosis agents. *PLoS Genet* **2**:208–223. doi:10.1371/journal.pgen.0020021

Duron O, Binetruy F, Noel V, Cremaschi J, McCoy K, Arnathau C, Plantard O, Goolsby J, Perez De Leon AA, Heylen DJA, Raoul Van Oosten A, Gottlieb Y, Baneth G, Guglielmone AA, Estrada-Pena A, Opara MN, Zenner L, Vavre F, Chevillon C. 2017. Evolutionary changes in symbiont community structure in ticks. *Mol Ecol* **26**:2905–2921. doi:10.1111/mec.14094

Duron O, Morel O, Noël V, Buysse M, Binetruy F, Lancelot R, Loire E, Ménard C, Bouchez O, Vavre F, Vial L. 2018. Tick-bacteria mutualism depends on B vitamin synthesis pathways. *Curr Biol* **28**:1–7. doi:10.1016/j.cub.2018.04.038

Elbir H, Almathen F, Alhumam NA. 2019. A glimpse of the bacteriome of *Hyalomma dromedarii* ticks infesting camels reveals human Helicobacter pylori pathogen. *J Infect Dev Ctries* **13**:1001–1012. doi:10.3855/JIDC.11604

Elbir H, Almathen F, Elnahas A. 2020. Low genetic diversity among *Francisella*-like endosymbionts within different genotypes of *Hyalomma dromedarii* ticks infesting camels in Saudi Arabia. *Vet World* **13**:1462–1472. doi:10.14202/vetworld.2020.1462-1472

Epis S, Sassera D, Beninati T, Lo N, Beati L, Piesman J, Rinaldi L, McCoy KD, Torina A, Sacchi L, Clementi E, Genchi M, Magnino S, Bandi C. 2008. *Midichloria mitochondrii* is widespread in hard ticks (Ixodidae) and resides in the mitochondria of phylogenetically diverse species. *Parasitology* **135**:485–494. doi:10.1017/S0031182007004052

Gerhart JG, Auguste Dutcher H, Brenner AE, Moses AS, Grubhoffer L, Raghavan R. 2018. Multiple acquisitions of pathogen-derived *Francisella* endosymbionts in soft ticks. *Genome Biol Evol* **10**:607–615. doi:10.1093/gbe/evy021

Ghoneim NH, Abdel-Moein KA, Zaher HM. 2017. Molecular detection of *Francisella spp*. among ticks attached to camels in Egypt. *Vector-Borne Zoonotic Dis* **17**:384–387. doi:10.1089/vbz.2016.2100

Ghosh S, Sela N, Kontsedalov S, Lebedev G, Haines LR, Ghanim M. 2020. An intranuclear *Sodalis*-like symbiont and *Spiroplasma* coinfect the carrot psyllid, *Bactericera trigonic*a (Hemiptera, Psylloidea). *microorganisms* **8**. doi: 10.3390/microorganisms8050692

Gillespie JJ, Drisco TP, Verhoeve VI, Utsuki T, Husseneder C, Chouljenko VN, Azad AF, MacAluso KR. 2014. Genomic diversification in strains of *Rickettsia* *felis* isolated from different arthropods. *Genome Biol Evol* **7**:35–56. doi:10.1093/gbe/evu262

Gillespie JJ, Joardar V, Williams KP, Driscoll T, Hostetler JB, Nordberg E, Shukla M, Walenz B, Hill CA, Nene VM, Azad AF, Sobral BW, Caler E. 2012. A *Rickettsia* genome overrun by mobile genetic elements provides insight into the acquisition of genes characteristic of an obligate intracellular lifestyle. *J Bacteriol* **194**:376–394. doi:10.1128/JB.06244-11

Gottlieb Y, Lalzar I, Klasson L. 2015. Distinctive genome reduction rates revealed by genomic analyses of two *Coxiella*-like endosymbionts in ticks. *Genome Biol Evol* **7**:1779–1796. doi:10.1093/gbe/evv108

Guizzo MG, Parizi LF, Nunes RD, Schama R, Albano RM, Tirloni L, Oldiges DP, Vieira RP, Oliveira WHC, Leite MDS, Gonzales SA, Farber M, Martins O, Vaz IDS, Oliveira PL. 2017. A *Coxiella* mutualist symbiont is essential to the development of *Rhipicephalus microplus*. *Sci Rep* **7**:1–10. doi:10.1038/s41598-017-17309-x

Ivanov IN, Mitkova N, Reye AL, Hübschen JM, Vatcheva-Dobrevska RS, Dobreva EG, Kantardjiev T V., Muller CP. 2011. Detection of new *Francisella*-like tick endosymbionts in *Hyalomma spp.* and *Rhipicephalus spp.* (Acari: Ixodidae) from Bulgaria. *Appl Environ Microbiol* **77**:5562–5565. doi:10.1128/AEM.02934-10

Ju JF, Bing XL, Zhao DS, Guo Y, Xi Z, Hoffmann AA, Zhang KJ, Huang HJ, Gong JT, Zhang X, Hong XY. 2020. *Wolbachia* supplement biotin and riboflavin to enhance reproduction in planthoppers. *ISME J* **14**:676–687. doi:10.1038/s41396-019-0559-9

Kurtti TJ, Felsheim RF, Burkhardt NY, Oliver JD, Heu CC, Munderloh UG. 2015. *Rickettsia buchneri* sp. nov., a rickettsial endosymbiont of the blacklegged tick *Ixodes scapularis*. *Int J Syst Evol Microbiol* **65**:965–970. doi:10.1099/ijs.0.000047

Larsson P, Oyston PCF, Chain P, Chu MC, Duffield M, Fuxelius HH, Garcia E, Hälltorp G, Johansson D, Isherwood KE, Karp PD, Larsson E, Liu Y, Michell S, Prior J, Prior R, Malfatti S, Sjöstedt A, Svensson K, Thompson N, Vergez L, Wagg JK, Wren BW, Lindler LE, Andersson SGE, Forsman M, Titball RW. 2005. The complete genome sequence of *Francisella tularensis*, the causative agent of tularemia. *Nat Genet* **37**:153–159. doi:10.1038/ng1499

Lin M, Zhang C, Gibson K, Rikihisa Y. 2009. Analysis of complete genome sequence of *Neorickettsia risticii*: Causative agent of Potomac horse fever. *Nucleic Acids Res* **37**:6076–6091. doi:10.1093/nar/gkp642

Lopes de Carvalho I, Toledo A, Carvalho CL, Barandika JF, Respicio-Kingry LB, Garcia-Amil C, García-Pérez AL, Olmeda AS, Zé-Zé L, Petersen JM, Anda P, Núncio MS, Escudero R. 2016. *Francisella* species in ticks and animals, Iberian Peninsula. *Ticks Tick Borne Dis* **7**:159–165. doi:10.1016/j.ttbdis.2015.10.009

Luo J, Liu MX, Ren QY, Chen Z, Tian ZC, Hao JW, Wu F, Liu XC, Luo JX, Yin H, Wang H, Liu GY. 2017. Micropathogen community analysis in *Hyalomma rufipes* via high-throughput sequencing of small RNAs. *Front Cell Infect Microbiol* **7**:1–12. doi:10.3389/fcimb.2017.00374

Nikoh N, Hosokawa T, Moriyama M, Oshima K, Hattori M, Fukatsu T. 2014. Evolutionary origin of insect-*Wolbachia* nutritional mutualism. *Proc Natl Acad Sci U S A* **111**:10257–10262. doi:10.1073/pnas.1409284111

Penz T, Schmitz-Esser S, Kelly SE, Cass BN, Müller A, Woyke T, Malfatti SA, Hunter MS, Horn M. 2012. Comparative genomics suggests an independent origin of cytoplasmic incompatibility in *Cardinium hertigii*. *PLoS Genet* **8**. doi:10.1371/journal.pgen.1003012

Perveen N, Muzaffar S Bin, Vijayan R, Al-Deeb MA. 2020. Microbial communities associated with the camel tick, Hyalomma dromedarii: 16S rRNA gene-based analysis. *Sci Rep* **10**:1–11. doi:10.1038/s41598-020-74116-7

Ravi A, Ereqat S, Al-Jawabreh A, Abdeen Z, Shamma OA, Hall H, Pallen MJ, Nasereddin A. 2018. Metagenomic profiling of ticks: Identification of novel rickettsial genomes and detection of tick borne canine parvovirus. *bioRxiv* 1–19. doi:10.1101/407510

Říhová J, Novaková E, Husník F, Hypša V. 2017. *Legionella* becoming a mutualist: Adaptive processes shaping the genome of symbiont in the louse *Polyplax serrata*. *Genome Biol Evol* **9**:2946–2957. doi:10.1093/gbe/evx217

Rio RVM, Symula RE, Wang J, Lohs C, Wu Y neng, Snyder AK, Bjornson RD, Oshima K, Biehl BS, Perna NT, Hattori M, Aksoy S. 2012. Insight into the transmission biology and species-specific functional capabilities of Tsetse (Diptera: Glossinidae) obligate symbiont *Wigglesworthia*. *MBio* **3**:1–13. doi:10.1128/mBio.00240-11

Sait M, Aitchison K, Wheelhouse N, Wilson K, Lainson FA, Longbottom D, Smith DGE. 2013. Genome sequence of *Lawsonia intracellularis* strain N343, isolated from a sow with hemorrhagic proliferative enteropathy. *Genome Announc* **1**:1–2. doi:10.1128/genomeA.00027-13

Santos-Garcia D, Rollat-Farnier PA, Beitia F, Zchori-Fein E, Vavre F, Mouton L, Moya A, Latorre A, Silva FJ. 2014. The genome of *Cardinium* cBtQ1 provides insights into genome reduction, symbiont motility, and its settlement in *Bemisia tabaci*. *Genome Biol Evol* **6**:1013–1030. doi:10.1093/gbe/evu077

Sassera D, Lo N, Epis S, D’Auria G, Montagna M, Comandatore F, Horner D, Peretó J, Luciano AM, Franciosi F, Ferri E, Crotti E, Bazzocchi C, Daffonchio D, Sacchi L, Moya A, Latorre A, Bandi C. 2011. Phylogenomic evidence for the presence of a flagellum and cbb3 oxidase in the free-living mitochondrial ancestor. *Mol Biol Evol* **28**:3285–3296. doi:10.1093/molbev/msr159

Selmi R, Ben Said M, Mamlouk A, Ben Yahia H, Messadi L. 2019. Molecular detection and genetic characterization of the potentially pathogenic *Coxiella burnetii* and the endosymbiotic *Candidatus* Midichloria mitochondrii in ticks infesting camels (*Camelus dromedarius*) from Tunisia. *Microb Pathog* **136**:103655. doi:10.1016/j.micpath.2019.103655

Seshadri R, Paulsen IT, Eisen JA, Read TD, Nelson KE, Nelson WC, Ward NL, Tettelin H, Davidsen TM, Beanan MJ, Deboy RT, Daugherty SC, Brinkac LM, Madupu R, Dodson RJ, Khouri HM, Lee KH, Carty HA, Scanlan D, Heinzen RA, Thompson HA, Samuel JE, Fraser CM, Heidelberg JF. 2003. Complete genome sequence of the Q-fever pathogen *Coxiella burnetii*. *Proc Natl Acad Sci U S A* **100**:5455–5460. doi:10.1073/pnas.0931379100

Sjödin A, Svensson K, Öhrman C, Ahlinder J, Lindgren P, Duodu S, Johansson A, Colquhoun DJ, Larsson P, Forsman M. 2012. Genome characterisation of the genus *Francisella* reveals insight into similar evolutionary paths in pathogens of mammals and fish. *BMC Genomics* **13**. doi:10.1186/1471-2164-13-268

Smith TA, Driscoll T, Gillespie JJ, Raghavan R. 2015. A *Coxiella*-like endosymbiontis a potential vitamin source for the lone star tick. *Genome Biol Evol* **7**:831–838. doi:10.1093/gbe/evv016

Szigeti A, Kreizinger Z, Hornok S, Abichu G, Gyuranecz M. 2014. Detection of *Francisella*-like endosymbiont in *Hyalomma rufipes* from Ethiopia. *Ticks Tick Borne Dis* **5**:818–820. doi:10.1016/j.ttbdis.2014.06.002

Visser ES, McGuire TC, Palmer GH, Davis WC, Shkap V, Pipano E, Knowles DP. 1992. The *Anaplasma marginale* *msp5* gene encodes a 19-kilodalton protein conserved in all recognized *Anaplasma* species. *Infect Immun* **60**:5139–5144. doi:10.1128/iai.60.12.5139-5144.1992

Wang Y, Mao L, Sun Y, Wang Z, Zhang Jiayong, Zhang Jibo, Peng Y, Xia L. 2018. A novel *Francisella*-like endosymbiont in *Haemaphysalis longicornis* and *Hyalomma asiaticum*, China. *Vector-Borne Zoonotic Dis* **18**:669–676. doi:10.1089/vbz.2017.2252
